# Targeting Phosphatidylserine Synthesis for Tumor Cell Suppression in Esophageal Squamous Cell Carcinoma and Glioblastoma

**DOI:** 10.3390/ijms27146226

**Published:** 2026-07-13

**Authors:** Yixuan Hu, Yaqi Cui, Zeqiong Xu, Duo Wu, Xiaochun Yu

**Affiliations:** 1 State Key Laboratory of Gene Expression, School of Life Sciences, Westlake University, Hangzhou 310030, China; 2 Westlake Laboratory of Life Sciences and Biomedicine, Hangzhou 310024, China

**Keywords:** PTDSS1, phosphatidylserine, lipid metabolism, esophageal squamous cell carcinoma, glioblastoma

## Abstract

While altered lipid metabolism is a hallmark of cancer, specific phospholipid dependencies remain poorly defined. Here, we identify phosphatidylserine synthase 1 (PTDSS1) as a targetable metabolic vulnerability in esophageal squamous cell carcinoma (ESCC) and glioblastoma (GBM). Pharmacological inhibition of PTDSS1 selectively and potently suppresses tumor growth both in vitro and in vivo. Mechanistically, PTDSS1 blockade triggers a rapid collapse of cellular phosphatidylserine (PS) and phosphatidylethanolamine (PE) pools, fundamentally disrupting endoplasmic reticulum (ER) homeostasis. This targeted lipid depletion activates a PERK-mediated autophagic response that ultimately yields to apoptosis. Clinically, pan-cancer transcriptomic analysis links elevated *PTDSS1* expression to reduced overall survival across diverse malignancies. Collectively, we establish PTDSS1 as an essential maintainer of ER integrity and highlight PS biosynthesis as a viable therapeutic target for exploiting tumor-specific metabolic dependencies.

## 1. Introduction

While altered glucose and central carbon metabolism are classically defined features of cancer, the reprogramming of membrane lipid biosynthesis is increasingly recognized as a critical dependency for tumor progression [1,2,3,4]. To sustain rapid proliferation, metastasis, and immune evasion, malignant cells require a continuous supply of structural phospholipids [5,6,7,8]. Beyond mere membrane biogenesis, these dynamic lipid networks orchestrate essential oncogenic signal transduction and dictate membrane fluidity [9]. Consequently, exploiting the unique phospholipid dependencies of cancer cells represents a highly targeted, yet underexplored, therapeutic frontier.

As the most negatively charged glycerophospholipid in eukaryotic membranes [10], phosphatidylserine (PS) has garnered increasing attention as a potential therapeutic target [11,12,13,14,15,16]. Beyond its structural function, PS also acts as a key scaffold for signal transduction pathways that drive tumor growth and survival [17,18]. In mammals, PS is generated by two membrane-associated enzymes, phosphatidylserine synthase 1 (PTDSS1) and phosphatidylserine synthase 2 (PTDSS2). These two enzymes share little homology and have different mechanisms of action to produce PS [19,20]. While PTDSS1 is able to use either phosphatidylcholine (PC) or phosphatidylethanolamine (PE) with L-serine to synthesize PS, PTDSS2 is restricted to using PE for the generation of PS [21,22]. Crucially, while the simultaneous deletion of both *Ptdss1* and *Ptdss2* is embryonically lethal, single-knockout mice deficient in either isoform develop normally, retain fertility, and exhibit a normal lifespan [23]. This physiological robustness provides a critical therapeutic safety window, suggesting that targeting a specific PSS isoform can disrupt pathological pathways in tumors without compromising essential physiological homeostasis.

The therapeutic rationale for targeting PTDSS1 in malignancy is grounded in distinct biological vulnerabilities. Historically, clinical interest focused on a subset of tumors harboring genetic deletions of *PTDSS2* (at 11p15.5), rendering them strictly dependent on PTDSS1 via a mechanism of synthetic lethality [24]. However, despite this clinical prevalence, naturally occurring *PTDSS2*-null cell lines are rare, limiting preclinical investigation [24]. This scarcity necessitates exploring PTDSS1 dependency in a broader context. Notably, recent studies have demonstrated that PTDSS1 activity is indispensable for the survival of B-cell lymphomas both in vitro and in vivo, even in the presence of functional PTDSS2 [25]. This critical observation implies that PTDSS1 dependency is not solely defined by genetic deletion but also extends to PTDSS2-intact malignancies, likely driven by the elevated metabolic demands characteristic of aggressive tumorigenesis.

Despite these compelling biological vulnerabilities, pharmacological targeting of PTDSS1 has only recently become feasible with the development of potent, selective small-molecule inhibitor DS55980254 (hereafter referred to as PTDSS1i), which was utilized in this study [24]. While these agents demonstrated robust efficacy in PTDSS2-deleted xenografts, their therapeutic potential in aggressive solid tumors remains largely unexplored. To bridge this gap, we prioritized two distinct models driven by metabolic necessity rather than genetic deletion. First, we selected esophageal squamous cell carcinoma (ESCC), where untargeted metabolomics identified glycerophospholipid metabolism as a top dysregulated pathway, with PTDSS1 serving as a significantly upregulated diagnostic biomarker [26]. Second, we focused on glioblastoma (GBM) due to its unique tissue-of-origin context: the mammalian brain is exceptionally enriched in PS (up to 15% of the phospholipid pool), which plays key roles in neurotransmission, synaptic plasticity, and neuroinflammation [27]. GBM cells are hypothesized to inherit a high baseline requirement for PTDSS1-driven synthesis to maintain membrane integrity within this lipid-rich niche. We postulate that this intrinsic lipid biosynthetic burden renders both tumor types exceptionally vulnerable to PTDSS1 inhibition.

In this study, we identify PTDSS1 as an actionable metabolic vulnerability in ESCC and GBM. We demonstrate that PTDSS1 is aberrantly upregulated in these malignancies and that its pharmacological inhibition selectively impairs tumor proliferation in vitro. Mechanistically, PTDSS1 blockade disrupts the PS-PE metabolic axis, provoking severe ER stress and a compensatory autophagic response that precedes terminal apoptosis. Consistently, blockade of PTDSS1 significantly suppresses xenograft growth and extends survival in vivo without eliciting overt systemic toxicity. Collectively, our work delineates a strict PS-dependent metabolic requirement in these solid tumors, validating PTDSS1 inhibition as a viable avenue for anticancer intervention.

## 2. Results

### 2.1. Aberrant Upregulation of PTDSS1 in ESCC and GBM Correlates with Poor Clinical Outcomes

**Figure 1:**
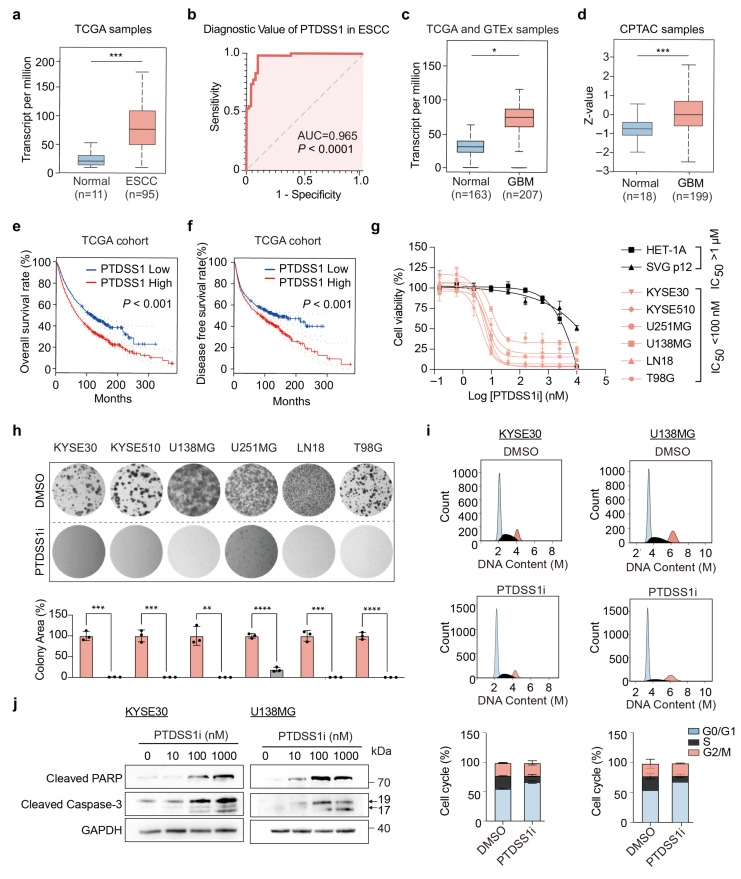
PTDSS1 is overexpressed in ESCC and GBM, and its inhibition suppresses tumor growth in vitro. (**a**). Elevated *PTDSS1* mRNA levels in ESCC tumors (*n* = 95) versus normal tissues (*n* = 11) from the TCGA dataset. (**b**). ROC analysis for ESCC diagnosis in the GSE23400 cohort (AUC = 0.965, *p* < 0.0001). (**c**). Box plot showing elevated PTDSS1 mRNA expression in TCGA GBM tumors (*n* = 207) compared with GTEx normal brain tissues (*n* = 163). (**d**). Increased PTDSS1 protein abundance in the CPTAC GBM cohort (Tumor *n* = 199, Normal *n* = 18; *p* < 0.001). (**e**,**f**) Kaplan–Meier analysis of (**e**) Overall survival (OS) and (**f**) disease-free survival (DFS) in the TCGA pan-cancer cohort. High *PTDSS1* expression correlates with poor prognosis (*p* < 0.001). (**g**). Dose–response curves of various human cancer and immortalized cell lines treated with PTDSS1i for 7 days. Cell lines are categorized into sensitive (IC_50_ < 100 nM, red) and resistant (IC_50_ > 1 µM, black) groups (*n* = 3). (**h**). Representative images and quantification of colony formation assays illustrating the abrogation of clonogenic survival in ESCC and GBM cell lines following long-term PTDSS1i exposure (*n* = 3). (**i**). Flow cytometric analysis of cell cycle distribution in KYSE30 and U138MG cells following treatment with DMSO or 100 nM PTDSS1i (*n* = 3). Top: representative histograms; bottom: quantitative percentage of cells in G0/G1, S, and G2/M phases. (**j**). Immunoblot analysis of cleaved PARP and cleaved Caspase-3, indicating dose-dependent induction of apoptosis following PTDSS1i treatment. Data are presented as mean ± SD from at least three independent experiments. * *p* < 0.05, ** *p* < 0.01, *** *p* < 0.001, **** *p* < 0.0001 by two-tailed Student’s *t*-test (**a**–**d**,**h**,**i**) or log-rank test (**e**,**f**).

To evaluate the clinical relevance of *PTDSS1*, we first examined its expression patterns in two distinct aggressive malignancies: ESCC and GBM. Analysis of the TCGA-ESCC cohort revealed a marked elevation of *PTDSS1* mRNA in tumor tissues compared to normal controls (Figure 1a), a finding further supported by its high diagnostic accuracy in the independent GSE23400 dataset (AUC = 0.965, *p* < 0.0001; Figure 1b). Parallel to our observations in ESCC, we found that *PTDSS1* was similarly overexpressed in GBM across integrated TCGA and GTEx datasets (Figure 1c). To confirm whether this widespread transcriptional upregulation leads to increased protein abundance, we further analyzed the CPTAC-GBM proteomic cohort. Consistent with the mRNA data, PTDSS1 protein levels were significantly higher in GBM tumors than in normal brain tissues (*p* < 0.001; Figure 1d). These results underscore a consistent oncogenic activation of *PTDSS1* across both ESCC and GBM at multiple molecular levels. Finally, to assess the broader impact of *PTDSS1*, we performed pan-cancer survival analysis, which revealed that high *PTDSS1* expression significantly correlates with shortened overall survival (OS) and disease-free survival (DFS) across diverse cancer types (Figure 1e,f).

### 2.2. Pharmacological Inhibition of PTDSS1 Suppresses Cell Proliferation and Induces Apoptosis in ESCC and GBM Cells

To determine whether the clinical prevalence of PTDSS1 translates into a functional metabolic dependency, we profiled the sensitivity of a diverse panel of malignant and non-transformed cell lines to PTDSS1i, a potent and selective small-molecule inhibitor of PTDSS1. HET-1A and SVG p12 were utilized as immortalized, non-malignant counterparts for the esophagus and brain respectively to rigorously evaluate the therapeutic selectivity of PTDSS1 inhibition across tissue-specific models. Cell viability assays (CTG) conducted over a 7-day period revealed that PTDSS1i exhibited low nanomolar potency in ESCC (KYSE30, KYSE510) and GBM (U251MG, U138MG, T98G, and LN18) lineages, with half-maximal inhibitory concentrations (IC_50_) ranging from 4.3 to 10.9 nM (Figure 1g). Notably, the non-cancerous HET-1A and SVG p12 cells exhibited remarkable resistance to PTDSS1 inhibition (IC_50_ > 1 µM), suggesting a favorable safety profile and a potentially wide therapeutic window for PTDSS1i (Figure 1g). Consistently, basal transcriptional profiling revealed that sensitive tumor cells exhibit a profoundly elevated *PTDSS1*/*PTDSS2* mRNA ratio compared to resistant non-transformed cells (Supplementary Scheme S1a,b). This basal *PTDSS2* pool in normal tissues likely provides a compensatory reserve to survive PTDSS1 blockade. These results were further corroborated by long-term colony formation assays, which demonstrated that PTDSS1 blockade effectively abolished the clonogenic potential of sensitive ESCC and GBM cells (Figure 1h).

To delineate the cellular mechanisms underlying this growth suppression, we analyzed cell cycle progression and cell death pathways. Flow cytometric analysis demonstrated that PTDSS1i treatment precipitated a distinct G0/G1 phase arrest in both representative ESCC (KYSE30) and GBM (U138MG) models, accompanied by a commensurate contraction of the S phase populations (Figure 1i). Furthermore, immunoblot analysis revealed a dose-dependent accumulation of the apoptotic markers cleaved PARP and cleaved Caspase-3 following PTDSS1i exposure (Figure 1j). Collectively, these data indicate that the pharmacological disruption of PTDSS1 suppresses tumor cell viability through a dual mechanism involving both the blockade of cell cycle progression and the induction of apoptosis.

### 2.3. PTDSS1 Inhibition Enforces a Selective Collapse of the PS-PE Metabolic Axis

**Figure 2:**
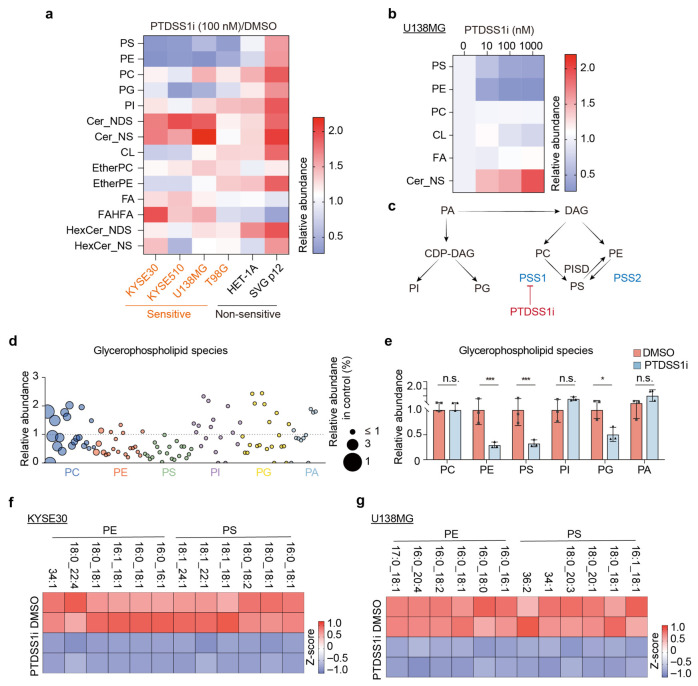
PTDSS1 inhibition enforces a selective collapse of the PS-PE metabolic axis. (**a**). Major lipid alterations in different tumor cells following treatment with DMSO or 100 nM PTDSS1i for 72 h. Data are presented as a heatmap of the percentage of vehicle control. (**b**). Dose-dependent lipidomic changes in U138MG cells treated with varying concentrations of PTDSS1i. (**c**). Schematic representation of major glycerophospholipid metabolism. Black arrows indicate the direction of metabolic flux. Blue text represents key PS synthetic enzymes, and the red text accompanied by a perpendicular line denotes the pharmacological blockade of PTDSS1 by PTDSS1i. (**d**). Fold-change analysis of individual glycerophospholipid species following treatment with DMSO or PTDSS1i. Circle size indicates the abundance of each of 100 phospholipids detected in control cells. Data are presented as the mean of three groups. (**e**). Bar graph showing changes in various glycerophospholipids in KYSE30 cells treated with DMSO or 100 nM PTDSS1i (mean ± SD of three independent experiments). (**f**,**g**). Heatmaps depicting the relative abundances (Z-score normalized) of specific PE and PS lipid molecules in KYSE30 (**f**) and U138MG (**g**) cells following PTDSS1i treatment. Statistical significance was determined using two-way ANOVA followed by Sidak’s multiple comparisons test. * *p* < 0.05, *** *p* < 0.001; n.s., not significant.

To elucidate the metabolic basis of the observed growth arrest, we performed untargeted lipidomics profiling on PTDSS1i-sensitive versus resistant cells. This revealed a dose-dependent restructuring of the lipid landscape in sensitive tumor cells (Figure 2a,b). Given PTDSS1’s canonical function in PS biosynthesis via PC base-exchange (Figure 2c), we interrogated the homeostasis of specific glycerophospholipid classes. Crucially, visualization of individual glycerophospholipid species uncovered that this metabolic perturbation was not stochastic; rather, it was driven by a widespread and synchronized depletion of the PS and PE repertoires (Figure 2d). In contrast, the abundances of other major phospholipid classes, including PC and phosphatidylinositol (PI) remained preserved around baseline levels, underscoring the high on-target selectivity of PTDSS1i (Figure 2d). Quantitative assessment confirmed that total PS and PE content exhibited a precipitous 70% reduction in sensitive ESCC cells. (Figure 2e). Mechanistically, the concomitant depletion of PE is attributable to the blockade of metabolic flux into the phosphatidylserine decarboxylase (PISD) pathway, which canonically converts PS to PE within the mitochondria (Figure 2c). Conversely, inhibitor-resistant cell lines displayed remarkable metabolic resilience, maintaining stable PS and PE homeostasis even under drug exposure (Figure 2a).

To address the possibility of acyl-chain-specific compensatory remodeling, we evaluated the fatty acyl compositions of the affected PE and PS species. Notably, in both ESCC (KYSE30) and GBM (U138MG) models, this downregulation was consistently observed across a broad spectrum of detected PE and PS molecules, largely irrespective of acyl chain length or degree of saturation (Figure 2f,g). Collectively, these data indicate that PTDSS1 blockade induces a broad depletion of the PS-PE axis, serving as a major biochemical event that likely contributes to the subsequent organelle stress in these malignancies.

### 2.4. Transcriptomic Profiling Reveals a Coordinated Program of ER Stress, Autophagy, and Apoptosis Induced by PS Depletion

**Figure 3:**
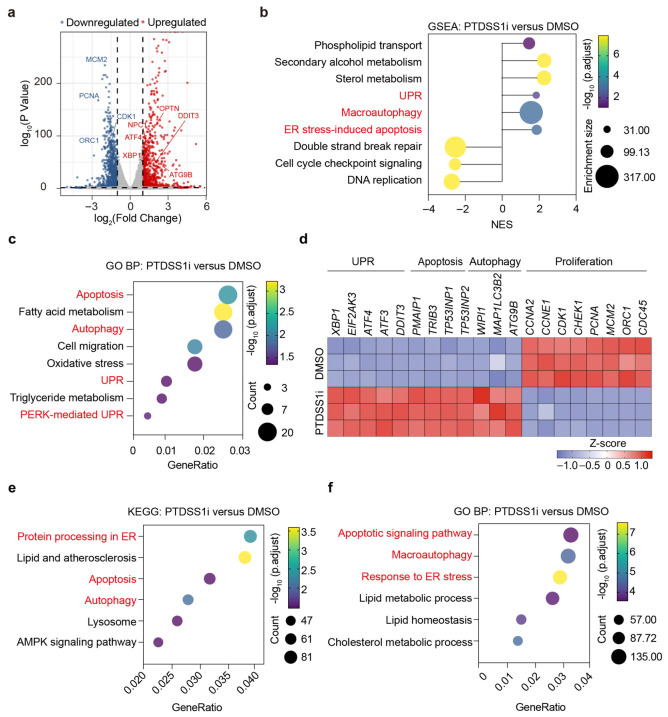
Transcriptomic profiling unveils a coordinated program of ER stress, autophagy, and apoptosis following PTDSS1 inhibition. (**a**). Volcano plot depicting global differential gene expression in KYSE30 cells treated with PTDSS1i versus DMSO control. Significantly upregulated (red) and downregulated (blue) genes are highlighted, with key transcripts involved in the UPR, autophagy, and cell cycle progression annotated. (**b**). GSEA of the KYSE30 transcriptome, illustrating positive normalized enrichment scores (NES) for UPR, macroautophagy, and ER stress-induced apoptosis, alongside negative enrichment for DNA replication and cell cycle checkpoints. (**c**). GO BP enrichment analysis of significantly upregulated pathways in PTDSS1i-treated KYSE30 cells. (**d**). Heatmap illustrating the relative expression (Z-score normalized) of representative gene clusters associated with the UPR, apoptosis, autophagy, and proliferation in KYSE30 cells. (**e**,**f**). KEGG pathway (**e**) and GO BP (**f**) enrichment analyses of differentially expressed genes in the U138MG GBM model treated with PTDSS1i. In panels (**b**,**c**,**e**,**f**), pathways highlighted in red text denote the key cellular stress responses (UPR, autophagy, and apoptosis) focused on in this study.

To systematically interrogate the global cellular consequences of the PS-PE metabolic collapse, we performed unbiased transcriptomic profiling (RNA-seq) on cells following PTDSS1 inhibition. In the ESCC model (KYSE30), volcano plot analysis revealed a dramatic transcriptional reprogramming. This shift was characterized by the induction of stress-responsive transcripts including key UPR sensors and autophagy initiators (*DDIT3*, *ATF4*, *XBP1*, *ATG9B*) and the concomitant severe suppression of genes driving cell cycle progression and DNA replication (e.g., *MCM2*, *PCNA*, *CDK1*) (Figure 3a).

To delineate the specific functional networks driving this response, we conducted Gene Set Enrichment Analysis (GSEA) and Gene Ontology Biological Process (GO BP) profiling in KYSE30 cells. These analyses demonstrated a significant positive enrichment for pathways associated with the UPR, PERK-mediated signaling, macroautophagy, and ER stress-induced apoptosis (Figure 3b,c). Inversely, gene sets essential for DNA replication, double-strand break repair, and cell cycle checkpoint signaling were depleted (Figure 3b). A targeted heatmap of core regulatory networks further confirmed this architectural shift, illustrating the synchronized upregulation of the UPR, autophagic, and apoptotic gene clusters, which inversely mirrored the collapse of the proliferative gene signature (Figure 3d).

Next, we sought to determine whether this metabolic stress signature was an ESCC-specific phenomenon or a conserved consequence of PTDSS1 blockade. Transcriptomic profiling of the GBM model (U138MG) corroborated a remarkably similar stress response. Both Kyoto Encyclopedia of Genes and Genomes (KEGG) and GO BP analyses in U138MG cells yielded near-identical enrichment profiles, highlighted by significant upregulation in UPR, macroautophagy, and apoptotic signaling pathways (Figure 3e,f). Collectively, these transcriptomic data suggest that the lipid perturbation inflicted by PTDSS1 blockade is likely coupled to ER stress, the mobilization of autophagy, and the engagement of apoptotic pathways.

### 2.5. PTDSS1 Blockade Precipitates ER Stress and Global Translational Repression

**Figure 4:**
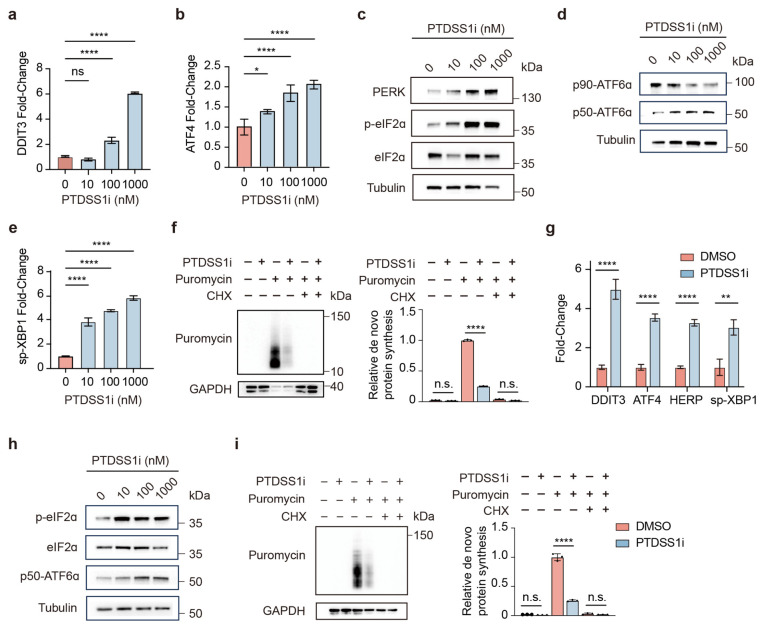
PTDSS1 blockade precipitates ER stress and global translational repression. (**a**–**f**) PTDSS1 inhibition induces UPR signaling and translational repression in KYSE30 cells. (**a**,**b**,**e**). Bar graphs showing dose-dependent increases in mRNA levels of (**a**) *DDIT3*, (**b**) *ATF4*, and (**e**) *sp-XBP1* assessed by RT-qPCR (*n* = 3). (**c**,**d**) Western blot analysis showing dose-dependent effects on UPR protein levels (PERK, p-eIF2a, and p50-ATF6a). (**f**). Surface sensing of translation (SUnSET) assay (left) and corresponding quantitative analysis of relative de novo protein synthesis (right) (*n* = 3). PTDSS1 inhibition severely represses global translation, mirroring the effect of the classical inhibitor CHX. Cells were treated with DMSO or 100 nM PTDSS1i for 48 h, followed by labeling with 10 µg/mL puromycin for 10 min. CHX was used as a positive control. (**g**–**i**) Validation of UPR activation and translational repression in U138MG cells. (**g**). Fold changes in mRNA expression levels of *DDIT3*, *ATF4*, *HERP*, and *sp-XBP1* (*n* = 3). (**h**). Western blot showing UPR pathway protein expression. (**i**). Representative SUnSET assay images and quantitative assessment demonstrating a profound shutdown of the cellular protein synthesis machinery (*n* = 3). Data are presented as mean ± SD from three independent experiments. * *p* < 0.05, ** *p* < 0.01, **** *p* < 0.0001 by two-tailed Student’s *t*-test; n.s., not significant.

To experimentally validate this ER crisis and dissect its molecular drivers, we next targeted the canonical UPR network, a stress surveillance system that monitors both protein folding capacity and membrane lipid homeostasis [28,29]. Consistent with our transcriptomic predictions, the interrogation of the UPR revealed a dose-dependent induction of hallmark effectors DDIT3 and ATF4 at the mRNA level in ESCC cells (Figure 4a,b). We further examined the three canonical branches of the UPR: PERK, IRE1a, and ATF6 [30]. PTDSS1i treatment triggered phosphorylation of eIF2a via the PERK branch (Figure 4c). Simultaneously, we observed the accumulation of cleaved p50-ATF6a and the IRE1a-mediated induction of *spliced-XBP1* (*sp-XBP1*), indicating the activation of the UPR network (Figure 4d,e). A critical downstream consequence of eIF2a phosphorylation is the attenuation of global protein synthesis. Utilizing the surface sensing of translation (SUnSET) assay, we demonstrated that PTDSS1 inhibition repressed de novo translation in ESCC cells, mirroring the profound blockade induced by cycloheximide (CHX) (Figure 4f).

To determine whether this ER crisis is a conserved mechanism across PTDSS1-dependent malignancies, we extended our analysis to the GBM model. Corroborating our findings in ESCC, PTDSS1 inhibition in U138MG cells elicited a parallel upregulation of UPR effectors, including *DDIT3*, *ATF4*, *HERP*, and *sp-XBP1* (Figure 4g). This was accompanied by the phosphorylation of eIF2a and ATF6 activation (Figure 4h), leading to a consequent global suppression of de novo protein synthesis (Figure 4i). Collectively, these findings establish that PTDSS1 is essential for maintaining ER function, and its pharmacological inhibition triggers an ER stress response that culminates in the shutdown of cellular protein machinery.

### 2.6. PTDSS1 Blockade Triggers a Compensatory, Cytoprotective Autophagic Response

**Figure 5:**
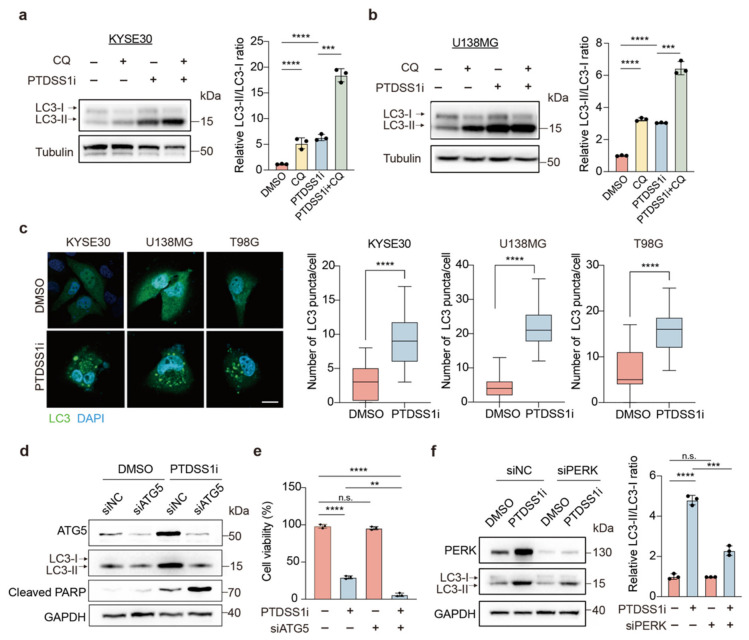
PTDSS1 blockade triggers a compensatory, cytoprotective autophagic flux that is partially mediated by PERK. (**a**,**b**) Autophagic flux analysis in KYSE30 (**a**) and U138MG (**b**) cells. Cells were treated with vehicle (DMSO) or 100 nM PTDSS1i for 48 h, with or without the lysosomal inhibitor chloroquine (CQ, 20 μM) added during the final 6 h. Immunoblots of LC3-I/II (left) and quantification of the LC3-II/LC3-I ratio (right) demonstrate the induction of de novo autophagic flux (*n* = 3). (**c**) Representative confocal immunofluorescence images (left) and box-plot quantification (right) of LC3 puncta formation in KYSE30, U138MG, and T98G cells following a 48h treatment with 100 nM PTDSS1i. Box plots display the median and interquartile range of puncta per cell (*n* = 50 cells per condition). Scale bar, 10 μm. (**d**,**e**) Genetic ablation of autophagy exacerbates PTDSS1i-induced apoptosis. KYSE30 cells were transfected with non-targeting control siRNA (siNC) or siRNA against ATG5 (siATG5) prior to 100 nM PTDSS1i exposure. Immunoblot analysis confirms ATG5 knockdown and shows enhanced PARP cleavage (**d**). Cell viability assays demonstrate significant sensitization to PTDSS1i-induced cytotoxicity following ATG5 depletion (**e**). (**f**) The autophagic response is partially dependent on PERK signaling. KYSE30 cells were transfected with siNC or siPERK followed by PTDSS1i treatment (*n* = 3). Immunoblotting (left) and quantification of the LC3-II/LC3-I ratio (right) reveal that PERK depletion significantly attenuates PTDSS1i-induced LC3 lipidation. Data are presented as mean ± SD from three independent experiments. Statistical significance was determined using one-way ANOVA with Tukey’s multiple comparisons test (**a**,**b**,**e**,**f**) or two-tailed Student’s *t*-test (**c**). ** *p* < 0.01, *** *p* < 0.001, **** *p* < 0.0001; n.s., not significant.

Autophagy functions as a central adaptive mechanism to mitigate ER stress and remodel damaged organelles [28,29]. Driven by the ER stress and transcriptomic shifts associated with PTDSS1 blockade, we investigated whether tumor cells mobilize this autophagic machinery as an adaptive response to the targeted lipid crisis. To distinguish de novo autophagic flux from a block in lysosomal clearance, we utilized the lysosomal inhibitor chloroquine (CQ). Immunoblot analysis in ESCC (KYSE30) and GBM (U138MG) models revealed that CQ co-treatment further augmented the PTDSS1i-induced accumulation of LC3-II compared to either treatment alone, confirming the induction of a complete autophagic flux (Figure 5a,b). This active autophagic state was corroborated by indirect immunofluorescence, which showed a redistribution of diffuse LC3 into cytosolic puncta following PTDSS1 inhibition (Figure 5c).

We then evaluated the functional consequence of this autophagic engagement by genetically ablating the core elongation factor ATG5 in KYSE30 cells (Figure 5d). Disruption of autophagy failed to rescue cells from PTDSS1i-induced cytotoxicity and instead sensitized them to the inhibitor. ATG5 depletion during PTDSS1i treatment exacerbated apoptotic cell death, evidenced by enhanced PARP cleavage (Figure 5d) and an accelerated loss of cell viability (Figure 5e). These data indicate that the autophagic response serves as a compensatory, cytoprotective mechanism deployed by tumor cells to maintain organelle homeostasis.

To define how the lipid depletion signal is transduced to the autophagic machinery, we focused on the ER stress triggered by PTDSS1 inhibition. We postulated that the PERK branch acts as a metabolic checkpoint, reprogramming the cell toward a cytoprotective autophagic state in response to the PS-PE imbalance. Silencing PERK expression in KYSE30 cells prior to PTDSS1i exposure significantly blunted the drug-induced LC3-I to LC3-II conversion (Figure 5f). Collectively, these data delineate a stress-response axis where PS and PE depletion induces ER stress, driving a compensatory autophagic flux that is significantly mediated by PERK. Ultimately, the progression to apoptosis indicates that this adaptive mechanism is insufficient to resolve the lipid crisis.

### 2.7. Exogenous PS Supplementation Rescues PTDSS1i-Induced ER Stress and Cytotoxicity

**Figure 6:**
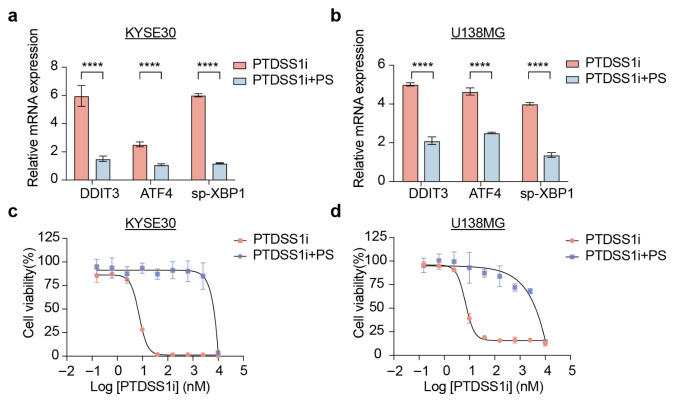
Exogenous PS supplementation rescues PTDSS1i-induced ER stress and cytotoxicity. (**a**,**b**) RT-qPCR analysis of UPR target genes *DDIT3*, *ATF4*, and *sp-XBP1* in KYSE30 (**a**) and U138MG (**b**) cells (*n* = 3). Cells were treated with 1 μM PTDSS1i alone or in combination with 80 μM PS liposomes for 48 h. Data are presented as mean ± SD of three independent experiments. **** *p* < 0.0001 by two-way ANOVA with multiple comparisons test. (**c**,**d**) Cell viability curves for KYSE30 (**c**) and U138MG (**d**) cells (*n* = 3). Cells were treated with varying concentrations of PTDSS1i in the presence or absence of 80 μM PS liposomes for 7 days. Data are presented as mean ± SD (*n* = 3).

To determine whether the observed ER stress and cytotoxicity are direct consequences of PS depletion, we performed a metabolic rescue experiment. Cells were supplemented with exogenous PS liposomes to bypass the PTDSS1 enzymatic blockade. PTDSS1i treatment induced the transcriptional upregulation of UPR markers DDIT3, ATF4, and sp-XBP1 in KYSE30 and U138MG cells. Concurrent administration of 80 μM PS liposomes reversed this induction (Figure 6a,b).

In prolonged cell viability assays, supplementation with exogenous PS protected tumor cells against cell death induced by PTDSS1 inhibition. As observed over a 7-day time course, PS addition remodeled dose–response curves and restored proliferative capacity (Figure 6c,d). These observations verify that both UPR activation and subsequent cytotoxicity upon PTDSS1 silencing are specific on-target effects arising from intracellular PS depletion.

### 2.8. Pharmacological Blockade of PTDSS1 Exhibits Potent In Vivo Anti-Tumor Efficacy

**Figure 7:**
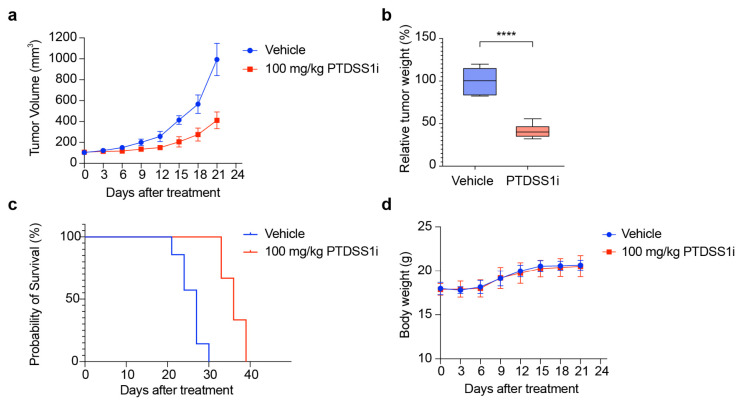
Pharmacological blockade of PTDSS1 exerts potent in vivo anti-tumor efficacy. (**a**). Tumor growth trajectories of KYSE30 xenografts in BALB/c nude mice treated with vehicle or PTDSS1i. Treatment was initiated when tumors reached an average volume of 100 mm^3^. Data are presented as mean ± SEM for 6 mice per group. (**b**). Relative tumor weight at the experimental endpoint on day 21. The center line indicates the median, box limits indicate upper and lower quartiles, and whiskers represent minimum and maximum values. **** *p* < 0.0001 by two-tailed Student’s *t*-test. (**c**). Kaplan–Meier survival analysis of mice bearing KYSE30 xenografts. PTDSS1i treatment prolonged overall survival as evaluated by the log-rank test. (**d**). Longitudinal monitoring of mouse body weights during the 21-day treatment period. Data are presented as mean ± SD.

To determine whether PTDSS1 dependency constitutes a viable therapeutic vulnerability in vivo, we established an ESCC xenograft model using KYSE30 cells. Once tumors reached approximately 100 mm^3^, mice were randomized to receive either vehicle or PTDSS1i. Consistent with our in vitro findings, PTDSS1 blockade significantly impaired tumor progression. Longitudinal monitoring revealed marked suppression of tumor growth kinetics in the PTDSS1i-treated cohort, in contrast to the rapid exponential expansion observed in vehicle-treated controls (Figure 7a). By the experimental endpoint, PTDSS1 inhibition had reduced terminal tumor weight by approximately 60% (Figure 7b).

Importantly, this targeted intervention conferred a substantial survival benefit. Kaplan–Meier analysis demonstrated that mice receiving PTDSS1i exhibited prolonged overall survival (Figure 7c). Moreover, the PTDSS1i regimen was well-tolerated. We observed no significant body weight loss or gross systemic toxicity throughout the study duration (Figure 7d), supporting a favorable therapeutic window where non-transformed tissues maintain systemic lipid homeostasis despite PTDSS1 inhibition. Collectively, these data validate PTDSS1 as an actionable metabolic target, demonstrating that exploiting this specific phospholipid dependency yields in vivo anti-tumor efficacy.

### 2.9. PTDSS1 Expression Serves as a Generalized Prognostic Indicator Across Multiple Human Malignancies

**Figure 8:**
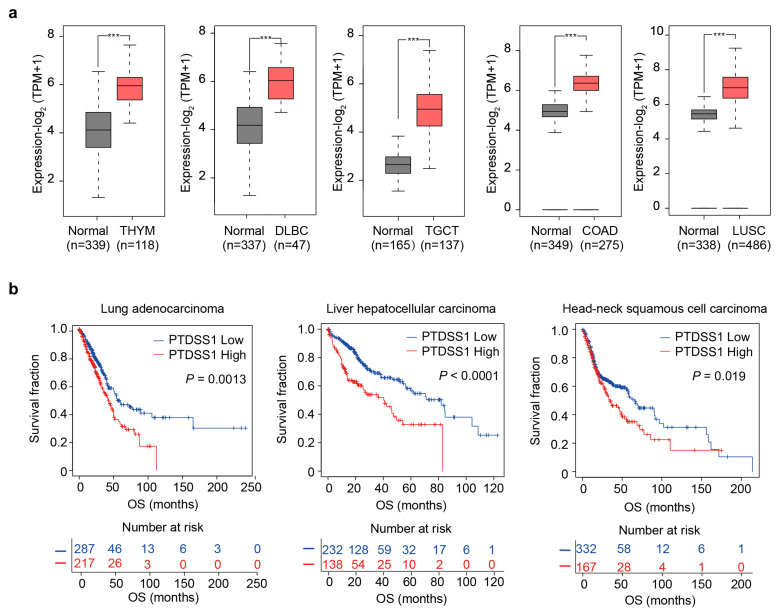
PTDSS1 expression serves as a generalized prognostic indicator across multiple human malignancies. (**a**). Box plots comparing *PTDSS1* mRNA expression levels in tumor tissues versus normal controls in thymoma (THYM), diffuse large B-cell lymphoma (DLBC), testicular germ cell tumors (TGCT), colon adenocarcinoma (COAD), and lung squamous cell carcinoma (LUSC). Data are derived from the TCGA and GTEx datasets. The center line represents the median, box limits indicate the upper and lower quartiles, and whiskers represent the min/max values. (**b**). Kaplan–Meier curves comparing overall survival between patients with high (red) and low (blue) *PTDSS1* expression in lung adenocarcinoma (LUAD), liver hepatocellular carcinoma (LIHC), and head and neck squamous cell carcinoma (HNSC). *** *p* < 0.001.

To evaluate the broader clinical relevance of our findings beyond ESCC and GBM, we performed an extensive pan-cancer analysis using integrated transcriptomic data from the TCGA and GTEx databases (Figure 8). Computational profiling revealed that PTDSS1 mRNA is significantly upregulated across a diverse spectrum of human cancers compared to their corresponding normal tissues, including thymoma (THYM), diffuse large B-cell lymphoma (DLBC), testicular germ cell tumors (TGCT), colon adenocarcinoma (COAD), and lung squamous cell carcinoma (LUSC) (Figure 8a).

We further investigated whether this aberrant expression translates into clinical outcomes. Kaplan–Meier survival analysis demonstrated that elevated PTDSS1 levels are strongly associated with diminished OS in several aggressive malignancies, most notably in lung adenocarcinoma, liver hepatocellular carcinoma, and head and neck squamous cell carcinoma (Figure 8b). Collectively, these pan-cancer data suggest that the PTDSS1-dependent metabolic axis is a conserved feature of malignant progression, identifying PTDSS1 as a promising and broadly applicable prognostic biomarker and therapeutic target in oncology.

## 3. Discussion

Our study identifies PTDSS1 as a targetable metabolic dependency in ESCC and GBM. We demonstrate that PTDSS1 blockade induces a specific collapse of the PS-PE metabolic axis, which is functionally coupled to ER stress, a compensatory autophagic response, and terminal apoptosis. Crucially, this stress cascade exploits the rigid biosynthetic demands of aggressive malignancies, providing a therapeutic window that spares non-transformed tissues.

A central mechanistic question arising from our findings is how a specific reduction in membrane lipids translates into the UPR signaling and cytotoxicity observed. Canonical UPR is typically driven by the luminal accumulation of misfolded proteins, while recent work has established lipid bilayer stress (LBS) as an alternative, independent upstream trigger of the UPR cascade [31,32,33]. PS and PE are indispensable structural lipids that govern ER membrane curvature and lateral lipid packing density [34]. We hypothesize that their depletion upon PTDSS1 blockade physically destabilizes the ER lipid bilayer, thereby engaging UPR transducers. However, we acknowledge that without direct biophysical assays evaluating membrane fluidity or lipid packing, this LBS-driven mechanism remains a speculative working model. Furthermore, this physical stress may be compounded by impaired ER calcium retention, as prior studies have linked shifts in phospholipid composition to disrupted SERCA-mediated calcium homeostasis [35].

Following the induction of ER stress, cells mobilize a PERK-dependent, cytoprotective autophagic response. As demonstrated by our ATG5 ablation studies, the engagement of this autophagic machinery serves as a critical survival mechanism to buffer against PTDSS1i-induced toxicity. While autophagy canonically recycles intracellular components to restore homeostasis, the irreversible enzymatic blockade of PTDSS1 strictly prevents the de novo synthesis of the essential PS headgroup. Consequently, the autophagic degradation of existing organelles fails to replenish the depleted aminophospholipid pools, rendering this compensatory response a fundamentally futile catabolic cycle. The persistence of this unresolvable lipid crisis ultimately exhausts cellular adaptive capacity, driving the UPR to transition toward terminal apoptotic signaling. This progression reflects an adaptive failure, where the severity of the PS-PE collapse simply overwhelms the cellular compensatory machinery.

Crucially, this targeted cytotoxicity is highly selective to tumor cells, sparing non-transformed counterparts. We attribute this therapeutic window to a disparity in baseline biosynthetic burden and isoform reliance. As supported by our transcriptional profiling, sensitive tumor cells exhibit a markedly elevated PTDSS1/PTDSS2 ratio, reflecting a strict dependency on PTDSS1 to sustain rapid membrane biogenesis. In this high demand state, the basal capacity of PTDSS2 is kinetically insufficient to compensate for PTDSS1 blockade. In contrast, non-transformed cells operate with lower proliferative demands; thus, the basal PS synthesis provided by PTDSS2 appears sufficient to maintain membrane integrity. PTDSS1 inhibition exploits a specific quantitative vulnerability in metabolic demand, where cancer cells lack the biosynthetic flexibility to survive the blockade.

In summary, our work establishes PTDSS1 as an essential maintainer of ER structural homeostasis, extending its known role beyond basal phospholipid biosynthesis. We demonstrate that the pharmacological blockade of PTDSS1 induces a targeted collapse of the PS-PE axis, which provokes ER stress and subsequently drives a futile catabolic cycle. By exploiting the rigid lipid biosynthetic demands of cancer cells, these findings validate the targeted disruption of PS biosynthesis as a highly specific and viable therapeutic strategy against aggressive solid tumors.

## 4. Materials and Methods

### 4.1. Cell Culture and Reagents

Human ESCC (KYSE30, KYSE510), GBM (U251MG, U138MG, T98G, LN-18), and control cell lines (HET-1A, and SVG p12) were obtained from the ATCC (Manassas, VA, USA) or JCRB (Ibaraki City, Japan) cell banks. Cells were all maintained at 37 °C in a humidified incubator with 5% CO_2_. Culture media were supplemented with 1% penicillin-streptomycin and fetal bovine serum (FBS) as follows: U251MG, U138MG, T98G and LN18 in DMEM; KYSE30 and KYSE510 in RPMI-1640/Ham’s F-12 (1:1); SVG p12 was cultured in Eagle’s Minimum Essential Medium (EMEM). HET-1A was cultured in Bronchial Epithelial Basal Medium (BEBM) supplemented with the BEGM™ SingleQuots™ Kit (Lonza, Basel, Switzerland). All cell lines were authenticated by STR profiling and routinely confirmed to be mycoplasma-free.

Key reagents included Chloroquine (MCE) and the PTDSS1 inhibitor DS55980254 (ProbeChem, Shanghai, China). Primary antibodies used for immunoblotting were: Anti-PERK (Proteintech, Rosemont, IL, USA, 20582-1-AP-50), anti-p-eIF2a Ser51 (Cell Signaling, Danvers, MA, USA, 3398), anti-eIF2a (Cell Signaling, 5324), anti-ATF6a (Proteintech, 24169-1-AP), anti-puromycin (Sigma-Aldrich, Macquarie Park, NSW, Australia, ZMS1016), anti-GAPDH (HUABIO, Woburn, MA, USA, ET1601-4), anti-b-tubulin (Proteintech, 10094-1-AP).

### 4.2. Animal Models

Animal experiments were conducted in accordance with protocols approved by Westlake University Animal Care and Use Committee. Six-week-old male BALB/c nude mice were obtained from Zhejiang Vital River Laboratory Animal Technology Co., Ltd. (Pinghu City, China) and housed under specific pathogen-free conditions.

To establish the ESCC xenograft model, 5 × 10^6^ KYSE30 cells suspended in 200 μL of a 1:1 mixture of PBS and Matrigel were injected subcutaneously into the right flank of each mouse. Once the average tumor volume reached approximately 100 mm^3^, mice were randomly assigned into two treatment groups (n = 12 per group). To evaluate both terminal tumor burden and long-term survival, each group was equally divided into two parallel sub-cohorts: an endpoint cohort (n = 6, sacrificed on day 21 for tumor weight and volume analysis) and a survival cohort (n = 6, monitored continuously). Mice received either vehicle or PTDSS1i at a dose of 100 mg/kg via daily oral gavage. The vehicle was formulated as a mixture of 5% DMSO, 40% PEG400, 20% of a 10% TPGS solution, and 35% H_2_O. Tumor dimensions were measured every 3 days using digital calipers, and tumor volumes were calculated using the formula V = 0.5 × length × width^2^. Body weights were monitored concurrently to assess systemic tolerability. For survival analysis, mice were euthanized when tumors reached a predefined surrogate volume endpoint of 1500 mm^3^ or upon exhibiting signs of severe morbidity.

### 4.3. Cell Viability and Colony Formation Assays

For viability assays, cells were seeded into 96-well plates (200 cells/well) and treated with DMSO or 100 nM PTDSS1i. The medium was refreshed every two days. On day 7, cell viability was quantified using CellTiter-Glo^®^ 2.0 (Promega, Madison, WI, USA) on a Varioskan LUX Microplate Reader (Thermo Fisher Scientific, Waltham, MA, USA). For colony formation assays, cells were seeded in six-well plates and treated with DMSO or 100 nM PTDSS1i for 7 days, with medium replenishment every 2–3 days. Colonies were fixed with 4% paraformaldehyde, stained with 0.1% crystal violet, and air-dried for imaging.

### 4.4. Cell Cycle Analysis

Cells treated with DMSO or 100 nM PTDSS1i for 48 h were harvested, washed with PBS, and fixed in 75% ethanol at –20 °C overnight. Fixed cells were subsequently washed with cold PBS, incubated with 10 μg/mL RNase A (Beyotime, Shanghai, China) at 37 °C for 30 min, and stained with 5 μg/mL propidium iodide (PI; eBioscience, San Diego, CA, USA) for 30 min at room temperature in the dark. DNA content distribution was analyzed using a CytoFLEX LX flow cytometer (Beckman, Lane Cove, NSW, Australia).

### 4.5. Western Blotting and Surface Sensing of Translation (SUnSET) Assay

To monitor global protein synthesis, a SUnSET assay was performed. Cells were treated with DMSO or 100 nM PTDSS1i for 48 h, labeled with 10 μg/mL puromycin (MCE) for 10 min, and harvested. For standard immunoblotting, cells were lysed in 2 × SDS buffer containing protease inhibitors (Beyotime, P1006) and phosphatase inhibitors (Roche, Basel, Switzerland, 4906845001), sonicated and boiled. Protein concentrations were normalized using the Pierce™ Rapid Gold BCA Assay. Lysates (20 μg) were separated by SDS–PAGE and transferred to PVDF membranes via the eBlot™ L2 system (GenScript, Piscataway, NJ, USA) for 15 min under the standard mode (90–120 V, 200–280 mA). Membranes were blocked with 5% skim milk for 1 h at room temperature and incubated with primary antibodies overnight at 4 °C, followed by HRP-conjugated secondary antibodies. For the SUnSET assay, puromycin incorporation was specifically detected using an anti-puromycin antibody (Sigma-Aldrich, ZMS1016, 1:1000). Signals were detected with SuperSignal™ West Pico PLUS chemiluminescent substrate (Thermo Fisher Scientific, 34580) and imaged with a ChemiDoc™ XRS+ system (Bio-Rad, Gladesville, NSW, Australia).

### 4.6. Lipidomics Analysis

Lipids were extracted using a modified MTBE/methanol protocol. Briefly, 1 × 10^7^ cells were harvested, washed, and resuspended in 1.5 mL methanol and 5 mL MTBE, then agitated for 1 h at room temperature. Phase separation was induced with 1.25 mL MS-grade water followed by centrifugation at 4 °C, 1000× *g* for 10 min. The upper organic layer was collected, and the lower aqueous phase was re-extracted with 2 mL MTBE/methanol/water (10:3:2.5, *v*/*v*/*v*). All combined extracts were dried under nitrogen gas and redissolved in 200 μL dichloromethane/methanol (3:1, *v*/*v*) for subsequent detection.

Lipid profiling was performed using an Agilent 1290 Infinity II UHPLC (Agilent Technologies, Santa Clara, CA, USA) coupled to a Bruker timsTOF Pro2 (Bruker Daltonics, Billerica, MA, USA). Separation was performed on a Waters ACQUITY BEH C18 column (Waters Corporation, Milford, MA, USA) (100 mm × 2.1 mm, 1.7 μm). Mobile phases A (water/methanol, 9:1) and B (acetonitrile/methanol/isopropanol, 2:3:5) were supplemented with 10 mM ammonium acetate and 0.2 mM ammonium fluoride. The 17 min elution gradient (flow rate, 0.3 mL/min; injection volume, 2 μL) was set as follows: 70% B (0–1 min), 70–86% B (1–3.5 min), 86% B (3.5–10 min), 86–100% B (10–11 min), 100% B (11–17 min). Data were acquired in positive and negative ESI modes (*m*/*z* 100–1350) with a TOF resolution ≥ 60,000. TIMS settings: 1/k0, 0.1–1.5 V·s/cm^3^; ramp time, 100 ms; accumulation time, 100 ms. ESI source: capillary voltage 4500 V, nebulizer 2 bar, dry gas 8 L/min (230 °C), sheath gas 4 L/min (400 °C).

### 4.7. RT-qPCR

Total RNA was extracted using TRIzol reagent (Invitrogen, Carlsbad, CA, USA). To remove genomic DNA, RNA was treated with gDNA wiper mix. First-strand cDNA was synthesized using HiScript IV RT SuperMix (Vazyme, Nanjing, China). Quantitative PCR was performed on a Bio-Rad CFX Connect system using Hieff^®^ qPCR SYBR Green Master Mix (YEASEN, Wuhan, China). The thermal cycling conditions were as follows: initial denaturation at 95 °C for 5 min, followed by 40 cycles of 95 °C for 10 s and 60 °C for 30 s. Melting curve analysis was conducted to confirm the specificity of PCR products. The relative gene expression was calculated using the 2^−ΔΔ*Ct*^ method. *GAPDH* was utilized as the internal reference gene for normalization. All RT-qPCR primer sequences and expected amplicon lengths are summarized in Supplementary Table S1.

### 4.8. Preparation of Phosphatidylserine Liposomes

Exogenous PS liposomes were prepared as previously described [36]. Briefly, PS dissolved in chloroform was evaporated to dryness under a stream of nitrogen gas to form a thin lipid film. The lipid film was resuspended in sterile serum-free medium to yield a 5 mM stock solution. To induce liposome formation, the suspension was sonicated on ice using a W-225R sonicator equipped with a 419 microprobe for three 5-min cycles. The resulting liposome suspension was filter-sterilized (0.22 μm) prior to cell treatment.

### 4.9. Immunofluorescence Staining

Cells grown on glass coverslips were treated with 100 nM PTDSS1i for 24 h. After washing with PBS, cells were fixed with 4% paraformaldehyde (PFA) for 15 min and permeabilized with 0.5% Triton X-100 for 5 min at room temperature. Following a 1 h blocking step with 5% BSA, samples were incubated with anti-LC3B primary antibody (1:200; CST, #2775) overnight at 4 °C. Cells were then stained with an Alexa Fluor 488-conjugated secondary antibody (1:500) for 1 h in the dark. Slides were mounted using DAPI-containing antifade medium (Vector Laboratories, Newark, CA, USA) and visualized using a Zeiss LSM 880 confocal microscope (Carl Zeiss, Jena, Germany). Quantitative analysis of LC3B puncta was performed using ImageJ software (version 1.54f), assessing at least 50 cells per condition.

### 4.10. Bioinformatic Analysis of Public Datasets

Transcriptomic and proteomic data were obtained from TCGA [37], GTEx [38], GEO (GSE23400) [39] and CPTAC [40]. PTDSS1 expression was analyzed in ESCC (TCGA: 95 tumors/11 normals) and GBM (TCGA: 207 tumors/GTEx: 163 normals; CPTAC: 199 tumors/18 normals). *PTDSS1* expression was also evaluated across THYM, DLBC, TGCT, COAD, and LUSC (tumor/normal *n*: 118/339, 47/337, 137/165, 275/349, 486/338). All bioinformatic analyses, including differential expression (Mann–Whitney *U* test) and prognostic evaluations (log-rank test), were executed and visualized using GEPIA2 [41].

### 4.11. Statistical Analysis

Data from *n* = 3 independent experiments are presented as mean ± SD. Statistical analyses were performed using GraphPad Prism 10.0. Two-group comparisons were evaluated by the two-tailed Student’s *t*-test (normally distributed data) or the Mann–Whitney *U* test (non-normally distributed data). *p* < 0.05 was considered significant.

## Data Availability

All data supporting the findings of this study are available within the paper and its Supporting Information. RNA-Seq data have been deposited in the Gene Expression Omnibus datasets under the Gene Expression Omnibus accession number GSE329207.

## Supplementary Materials

The following supporting information can be downloaded at: https://www.mdpi.com/article/10.3390/ijms27146226/s1.
