# Supplementary material for: Targeting Phosphatidylserine Synthesis for Tumor Cell Suppression in Esophageal Squamous Cell Carcinoma and Glioblastoma
Source: Int J Mol Sci. 2026 Jul 13;27(14):6226. doi: 10.3390/ijms27146226 (PMC13411466; doi:10.3390/ijms27146226)
Supplement: Supplementary file 1 [file ijms-27-06226-s001.zip › ijms-4349251-supplementary.pdf]

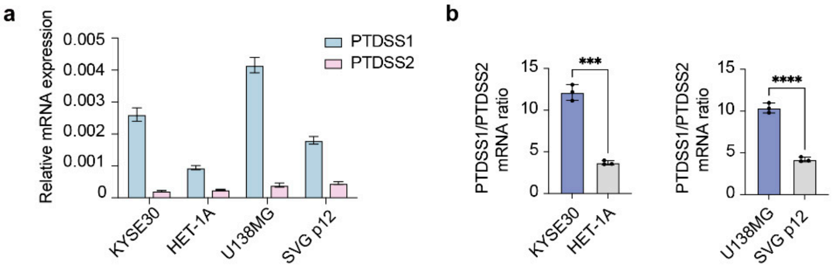

Scheme S1. Basal PTDSS1 and PTDSS2 expression profiles in tumor versus non-transformed cells.

(a) RT-qPCR analysis of basal PTDSS1 and PTDSS2 mRNA expression levels in representative ESCC (KYSE30), GBM (U138MG), and non-transformed (HET1A, SVGp12) cell lines. (b) The mRNA expression ratio of PTDSS1 to PTDSS2 calculated from the basal expression data. GAPDH was used as the internal reference gene for normalization. Data are presented as mean ± SD (n = 3 independent experiments). *** P < 0.001, **** P < 0.0001 by two-tailed Student’s t-test.

Supplementary Table S1. Oligonucleotide primer sequences used for quantitative real-time RT-PCR (RT-qPCR)

Gene	Forward primer (5' -> 3')	Reverse primer (5' -> 3')	Length
ATF4	GTTCTCCAGCGACAAGGCTA	ATCCTGCTTGCTGTTGTTGG	187
DDIT3	GGAAACAGAGTGGTCATTCCC	CTGCTTGAGCCGTTCATTCTC	116
Sp-XBP1	CTGAGTCCGAATCAGGTGCAG	ATCCATGGGGAGATGTTCTGG	145
Total XBP1	TGGCCGGGTCTGCTGAGTCCG	ATCCATGGGGAGATGTTCTGG	97
HERP	ATGGAGTCCGAGACCGAAC	TTGGTGATCCAACAACAGCTT	201
PTDSS1	GCAAGTGGAGGACATCACCAT	TCATCCCTGGTAAAGGCGAAG	105
PTDSS2	CTCACCTGTACGCTTGGCTAT	CCACAATACCTCTCTTGGTGTTG	82
GAPDH	GGAGCGAGATCCCTCCAAAAT	GGCTGTTGTCATACTTCTCATGG	197
